# Identification of a Neurocognitive Mechanism Underpinning Awareness of Chronic Tinnitus

**DOI:** 10.1038/s41598-017-15574-4

**Published:** 2017-11-09

**Authors:** Krysta J. Trevis, Chris Tailby, David B. Grayden, Neil M. McLachlan, Graeme D. Jackson, Sarah J. Wilson

**Affiliations:** 1 Melbourne School of Psychological Sciences, The University of Melbourne, Victoria, Australia; 2 The Florey Institute of Neuroscience and Mental Health, Melbourne, Victoria Australia; 3 Centre for Neural Engineering, Department of Electrical and Electronic Engineering, The University of Melbourne, Victoria, Australia

## Abstract

Tinnitus (ringing in the ears) is a common auditory sensation that can become a chronic debilitating health condition with pervasive effects on health and wellbeing, substantive economic burden, and no known cure. Here we investigate if impaired functioning of the cognitive control network that directs attentional focus is a mechanism erroneously maintaining the tinnitus sensation. Fifteen people with chronic tinnitus and 15 healthy controls matched for age and gender from the community performed a cognitively demanding task known to activate the cognitive control network in this functional magnetic resonance imaging study. We identify attenuated activation of a core node of the cognitive control network (the right middle frontal gyrus), and altered baseline connectivity between this node and nodes of the salience and autobiographical memory networks. Our findings indicate that in addition to auditory dysfunction, altered interactions between non-auditory neurocognitive networks maintain chronic tinnitus awareness, revealing new avenues for the identification of effective treatments.

## Introduction

Tinnitus is a form of phantom auditory perception, typically sensed as a ringing, buzzing, or hissing sound in the ears or head. There has been substantial progress in understanding the auditory mechanisms that generate tinnitus, including dysfunction in the peripheral and central auditory systems^1^. However, we do not know why tinnitus becomes chronic in some individuals but not others. The problem of chronic tinnitus is significant because it is a major public health issue associated with substantial economic and psychosocial burden for which there is no definitive cure^2,3^.

Investigations of the neural correlates of chronic tinnitus have indicated involvement of non-auditory regions, including the prefrontal, anterior cingulate and insula cortices, the amygdala, and the hippocampal formation^4,5^. This has led to the idea of a ‘tinnitus network’, typically characterized by altered auditory processing that generates the tinnitus sensation, which becomes associated with heightened attention and emotions (prefrontal cortex, anterior cingulate, insula, amygdala), consolidating memory of the sound (hippocampal region)^4,6^. By this account, the involvement of non-auditory brain regions in chronic tinnitus is viewed as a consequence or down-stream effect of the persistent tinnitus sensation, typically associated with its more pervasive effects on an individual’s psychosocial wellbeing^4^.

Based on behavioral evidence, we have recently shown that psychological markers of attention-switching, namely cognitive and emotional control, are impaired in chronic tinnitus suggesting reduced cognitive control may constitute a core mechanism that maintains the awareness of tinnitus, rather than being a down-stream effect^7^. Importantly, this finding remained after we eliminated potential factors that may influence cognitive control, such as the presence of a constant sound^8^, hearing difficulties^9^, tinnitus impact^10^, age^11^, and emotional wellbeing (i.e., depression, anxiety)^12,13^. Attention-switching is underpinned by efficient interactions between large-scale neurocognitive networks that facilitate processing of relevant stimuli, and inhibit goal-irrelevant stimuli. Specifically, the salience network (SN) directs dynamic network interactions, recruiting the cognitive control network (CCN) to direct attentional resources towards salient information and to down-regulate rumination and intrusive emotional experiences, associated with the affective (AN) and autobiographical memory networks (AMN)^7,14,15^. Impaired performance on tasks that rely on proficient CCN functioning has been observed in people with chronic tinnitus^10,16^, in addition to neuroimaging evidence suggesting dysfunction of prefrontal cortex in chronic tinnitus^4,17^, a core region of the CCN associated with cognitive control functioning^18^. Could persistent awareness of the tinnitus sensation result from dysfunction within the CCN?

Here, we interrogated the functioning of these established neurocognitive networks with a neuroimaging paradigm. We investigated whether altered functioning of the CCN underpins chronic tinnitus, as suggested by our behavioral work. To achieve this, 15 people with chronic tinnitus from the community and 15 healthy controls matched for age and gender performed a cognitively demanding task (the ‘*n*-back’ task), which is known to activate the CCN, while we measured blood oxygen level-dependent (BOLD) responses using functional magnetic resonance imaging (fMRI).

## Results

### Successful Activation of the Cognitive Control Network

**Figure 1:**
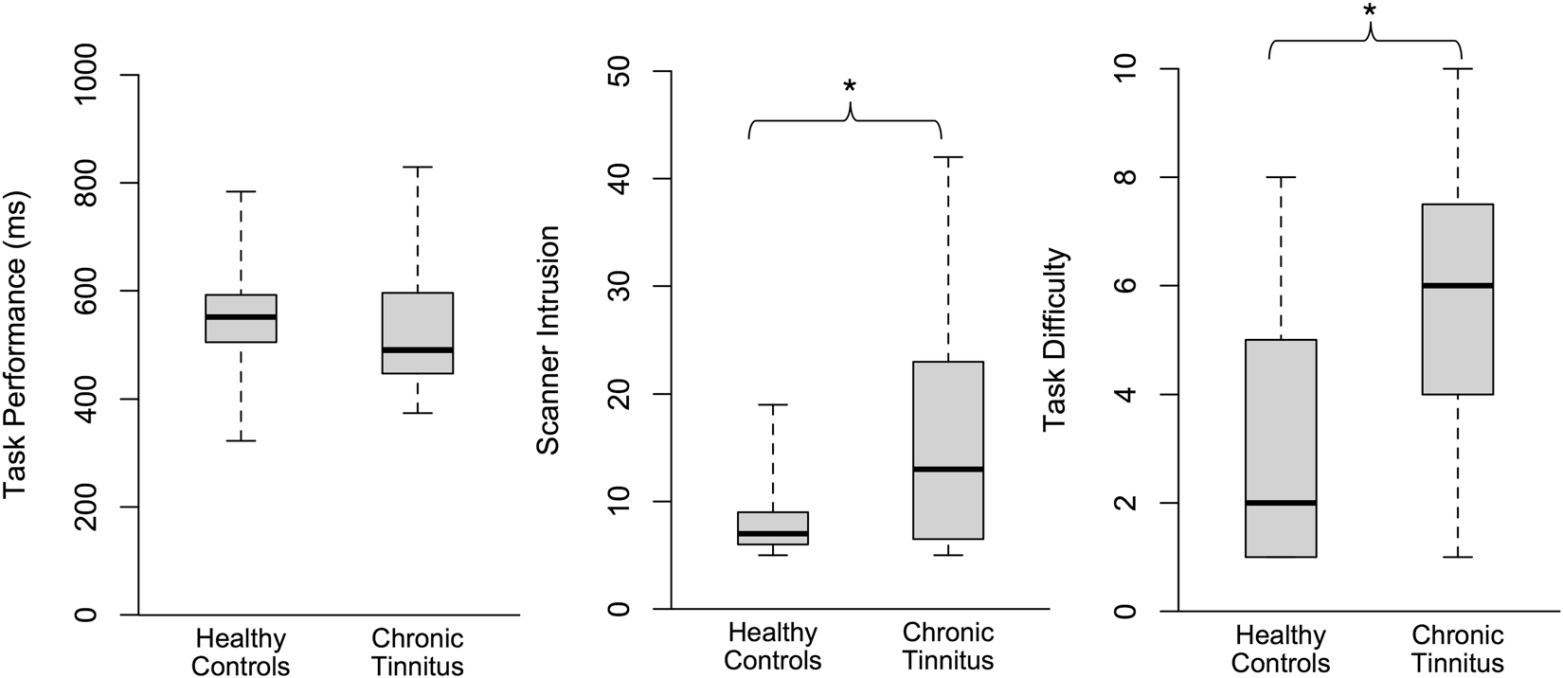
Behavioural experience of the *n*-back task in-scanner. Boxplots highlight the similarity in task performance between groups despite the chronic tinnitus group reporting significantly higher ratings of scanner intrusion and task difficulty compared to healthy controls, *p < 0.05.

Both groups performed similarly well on the *n*-back task during scanning, with no differences observed between the chronic tinnitus and healthy control groups in task accuracy or reaction times for the 0-back and 2-back conditions (all *p* > 0.500). Interestingly, despite similar objective performance on the task, the chronic tinnitus group reported that they found the task significantly more difficult than the healthy control group, *t*(28) = 2.23, *p* = 0.034, *d* = 1.27, and that the scanner noise was more intrusive, *t*(16.86) = 2.63, *p* = 0.018, Cohen’s *d* = 2.95 (Fig. 1). In addition, as both groups had hearing thresholds within the normal range and similarly minimal symptoms of anxiety or depression on the Hospital Anxiety and Depression Scale (HADS), it is most likely that group differences can be attributed to persistent awareness of the tinnitus sensation, which likely contributed to subjective task difficulty.

**Figure 2:**
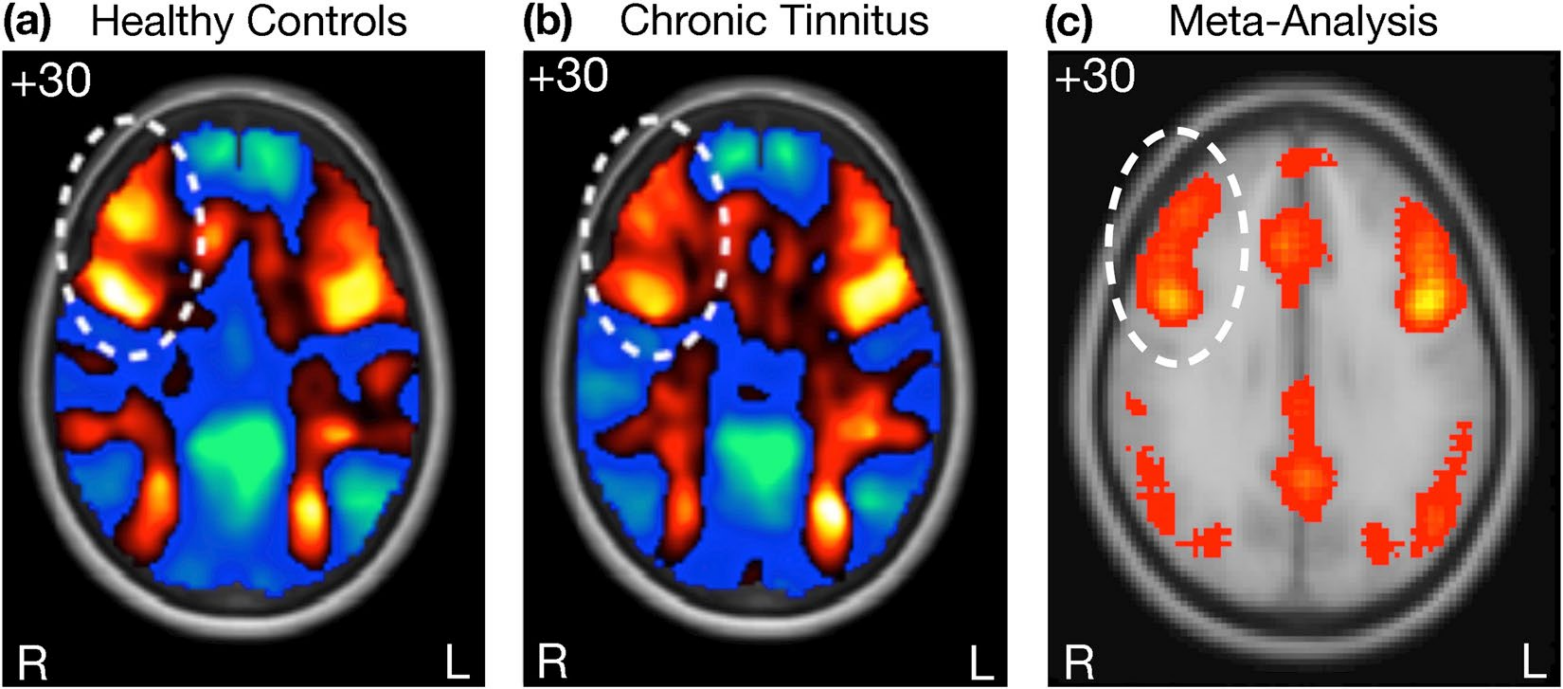
Activation of the Cognitive Control Network. (**a**), (**b**), SPM-T maps from the present study indicating activation of the CCN by the *n*-back task in the healthy control group (**a**) and the chronic tinnitus group (**b**), with the circled region suggesting reduced activation of the right middle frontal gyrus (rMFG) in the chronic tinnitus group. (**c)** We performed an automated reverse inference meta-analysis using NeuroSynth^67^ on 2633 fMRI studies that were identified using the search term ‘cognitive’. The results highlight broad network activation within nodes of the cognitive control network (CCN), anchored in bilateral dorsolateral prefrontal cortex and posterior parietal cortex^20^.

In line with successful performance of the *n*-back task, both the control and chronic tinnitus groups showed activation of the CCN when performing the task (Fig. 2). However, visual inspection suggested possible attenuated activation in chronic tinnitus in the right prefrontal cortex in the region of the right middle frontal gyrus (rMFG), a core node of the CCN (Fig. 2, circled region)^19^. Subtle disruptions to the functioning of a network node can lead to dysfunction of the network and its associations with other networks^20^. Could the functioning of this network node reflect more subtle disruption of the CCN in chronic tinnitus.

### Subtle Disruption of the Cognitive Control Network in Chronic Tinnitus

**Figure 3:**
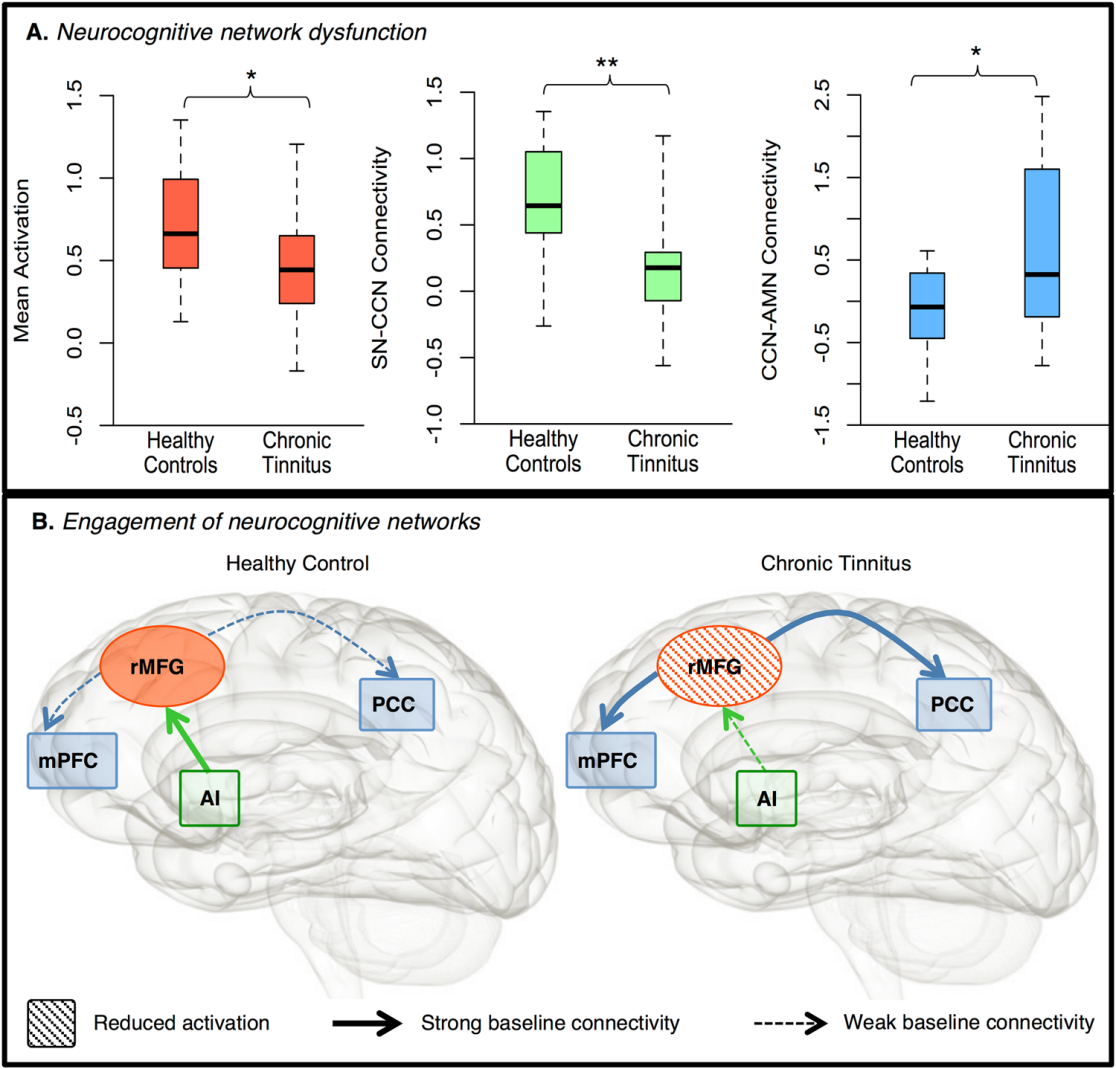
Functional deficits associated with dysfunction of the cognitive control network (CCN) in chronic tinnitus (*n* = 15) compared to healthy controls (*n* = 15). (**A**) *Neurocognitive network dysfunction:* Boxplots highlight significantly attenuated activation of the posterior right middle frontal gyrus (rMFG) during the *n*-back task in chronic tinnitus; significantly lower baseline connectivity between the right anterior insula (rAI) node of the salience network and the affected CCN node (rMFG) in chronic tinnitus; and significantly higher baseline connectivity between the affected CCN node (rMFG) and nodes of autobiographical memory network (AMN), including the left posterior cingulate cortex (PCC, illustrated here) and the left medial prefrontal cortex (mPFC). (**B**) *Engagement of neurocognitive networks*. Illustration of large-scale neurocognitive network functioning in healthy controls (left) and chronic tinnitus (right) with nodes of the CCN (red), SN (green) and AMN (blue). Here, while healthy controls show higher SN-CCN baseline connectivity associated with greater CCN activation, people experiencing chronic tinnitus show lower SN-CCN baseline connectivity and decreased CCN activation. This may underpin less proficient network switching, characterized by higher CCN-AMN baseline connectivity in chronic tinnitus, associated with difficulty switching attention away from the auditory environment (e.g. scanner noise), **p < 0.01; *p < 0.05.

To address this question, we specifically investigated the functioning of the rMFG using the second (independent) data set, ensuring noncircular analysis^21^. Here, we investigated both the activation of this CCN node and its baseline and task-dependent connectivity with nodes of other networks involved in attention-switching (SN, AMN), as well as their association with participant experience of in-scanner task performance. Replicating the findings of the first data set, we found significantly attenuated activation of the posterior rMFG in the chronic tinnitus group compared to the healthy controls *t*(28) = 2.06, *p* = 0.024, Cohen’s *d* = 0.46 (Fig. 3A). We also found reduced baseline connectivity in the chronic tinnitus group from a node of the SN, the right anterior insula (AI), to the affected CCN node (rMFG) *t*(28) = 3.03, *p* = 0.005, Cohen’s *d* = 0.74 (Fig. 3A), which was significantly correlated with the degree of reduced activation in the rMFG, *r*(30) = 0.43, *p* = 0.017, 95% BCa CI [0.13, 0.69]. No effect was found from the anterior cingulate node to the rMFG (*p* > 0.250).

In the AMN, we found increased baseline connectivity from the rMFG to core nodes of the AMN, namely the left posterior cingulate cortex (PCC) *t*(27.95) = 2.50, *p* = 0.019, *d* = 0.57 (Fig. 3A) and left medial prefrontal cortex (mPFC) *t*(22.69) = 2.33, *p* = 0.029, *d* = 0.81. The subjective ratings of the participants indicated that in comparison to healthy controls, the chronic tinnitus group found the *n*-back task significantly more challenging in the scanner than during practice, and reported the scanner noise as significantly more intrusive. Moreover, the increased CCN-AMN connectivity between the rMFG and left PCC node was significantly correlated with higher ratings of scanner noise intrusion during task performance, *r*(30) = 0.43, *p* = 0.019, 95% BCa CI [0.07, 0.71].

## Discussion

Together, these findings suggest that disrupted SN-CCN and CCN-AMN connectivity may have a core role in perpetuating awareness of chronic tinnitus. In particular, decreased SN-CCN connectivity may underpin dysfunction of the CCN and ineffective network-switching, characterised by increased CCN-AMN connectivity and the experience of greater difficulty engaging in goal-directed behavior (Fig. 3B). This was observed as greater difficulty switching attention away from the immediate auditory (stimulus-driven) environment (e.g. scanner noise) towards the cognitive requirements of the visual in-scanner task, suggesting that a failure of the SN to proficiently modulate CCN activity may facilitate persistent awareness of sensory sensations such as tinnitus, due to a failure to switch attention. This was reflected in attenuated CCN activation and dysfunctional connectivity between the SN, CCN and AMN, providing a possible mechanism for the ongoing psychological salience of the tinnitus sensation in people with chronic tinnitus.

While other work has found dysfunction in regions of the prefrontal cortex in chronic tinnitus^4,22^, our study is the first to use targeted activation of the CCN to interrogate its involvement in chronic tinnitus. Within the CCN, the rMFG is thought to modulate the transition between goal-directed (dorsal attention network) and stimulus-driven (ventral attention network) attention processes, thus reducing interference of irrelevant stimuli^23–25^. Dysfunction of this region, and the CCN more broadly, has been linked to increased distractibility, abnormal stimulus selection, and difficulty disengaging or ‘switching’ attention^24,26^. The anterior insula is thought to be a hub of self-awareness important for performance monitoring, sensory integration, and initiating switching between the CCN and AMN to achieve optimum performance^27,28^. The AMN, anchored in the mPFC and PCC, is associated with self-reflection, awareness and arousal, with the PCC proposed to facilitate meta-stability of large-scale neurocognitive networks for focused attention^15,29,30^. Other work has also found dysfunction in nodes of the SN and AMN in chronic tinnitus including the insula, PCC and mPFC identified here^5,31^. However, the present work demonstrates a specific mechanism by which the SN, CCN and AMN may contribute to the maintenance of chronic tinnitus. Specifically, altered functional connectivity between these networks may contribute to reduced proficiency in engaging the CCN and impaired down-regulation of internal awareness of the tinnitus sensation, maintaining its intrusiveness, psychological salience, and effects on emotional wellbeing. Moreover, dysfunction of these large-scale neurocognitive networks is associated with cognitive and affective features of a range of neurological and psychological disorders including chronic pain, depression and anxiety^20,32^. Specifically, hypoactivity of the CCN and reduced down-regulation of affective and autobiographic memory networks underpins symptoms of depression^33^, including reduced emotional wellbeing and increased rumination which frequently co-occur with chronic tinnitus^34^.

Further interrogation of SN, AMN and AN functioning in relation to the CCN disruption identified here will contribute to the development of more precise treatments to restore the functional balance within and between these networks. For example, neurofeedback training to improve network-switching, and optimised use of auditory and psychological therapies known to improve functioning of these networks^35–37^. Importantly, the SN, AN, and CCN are known to interact with the auditory system, particularly habituation of aversive sounds and top-down control of auditory processing^38–40^. Establishing how these networks interact with the auditory system from an attention-switching perspective will further elucidate how altered neurocognitive network function maintains awareness of this phantom auditory sensation.

Importantly, while *generation* of the tinnitus sensation is underpinned by peripheral and central auditory system dysfunction^1^, the present study identifies a potential mechanism *maintaining* awareness of tinnitus. Animal models emphasise the importance of hyperactivity in the peripheral auditory system, particularly the dorsal cochlear nucleus^41^ and inferior colliculus^42^, in generating the ‘bottom-up’ tinnitus percept^43^. Peripheral hyperactivity is considered a critical trigger for central auditory plasticity^44^ which, in turn, contributes to conscious perception of the tinnitus sensation^45^. In this context, the results of this study suggest ongoing perception of the tinnitus sensation may persist due to the failure of top-down mechanisms, such as attention-switching, to inhibit this bottom-up hyperactivity. In other words, dysregulation of salience and top-down control mechanisms, in addition to hyperactive bottom-up signals in the peripheral and central auditory system, may interact to perpetuate the tinnitus sensation. Future research investigating the dynamic interaction of top-down and bottom-up processes in human and animal models of tinnitus may further advance our understanding of how these functional network changes evolve. In particular, determining how neurocognitive network dysfunction develops will provide insight into the causes of reduced network integrity and, in turn, causes of chronic tinnitus and other psychological and neurological conditions.

Our sample was representative of chronic tinnitus in the general community, with all participants reporting constant tinnitus of presence, 67% reporting a mild impact of tinnitus on daily life, and approximately 50% awareness of the tinnitus sensation, suggesting some ability to mask this sensation in daily life. In contrast, clinical samples typically report a more severe impact on daily life and greater difficulty masking the sensation, commonly associated with increased psychological co-morbidity^46,47^. As such, replication of these results in a clinical cohort with severe tinnitus will add further insight into therapeutic targets to alleviate the impact of tinnitus on daily life. Longitudinal work may provide insight into the progression of network functioning from acute onset to chronic perception of the tinnitus sensation to elaborate on how the CCN, SN and AMN may interact to perpetuate the tinnitus sensation over time.

In conclusion, our results support the involvement of large-scale, non-auditory neurocognitive networks in maintaining awareness of chronic tinnitus, identifying impaired switching of attention as a neurocognitive mechanism underpinning this condition. Dysfunction of the cognitive control network is a feature of other neurological and psychological disorders with clinical features that overlap with the broad array of cognitive and affective symptoms of chronic tinnitus such as impaired cognitive and inhibitory control, attention, working and autobiographical memory, disturbed sleep, anxiety, somatization and depression^7,20,33,48–51^. As such, while tinnitus network theories fail to account for the heterogeneous presentation of people with chronic tinnitus, our results may offer an explanation for this broad array of cognitive and affective experiences through the functional imbalance of specific neurocognitive networks involved in attention-switching.

This systems neuroscience perspective signals a paradigm shift in our understanding of chronic tinnitus. It extends traditional auditory and tinnitus network accounts by considering the involvement of non-auditory regions as part of a mechanism, rather than a consequence, of chronic tinnitus. A reduction in the dynamic functioning of our ordinarily adaptive large-scale networks may perpetuate awareness of the tinnitus sound through a failure in attention-switching, a process that may also account for the pervasive psychological impact of chronic tinnitus. This approach provides a new platform for investigating the gap between the psychological experience of chronic tinnitus and its neurobiological underpinnings. New treatment strategies that aim to restore the functional balance within and between the specific networks involved in attention-switching (CCN, SN and AMN) may improve the health and wellbeing of people with chronic tinnitus.

## Methods and Materials

### Participants

We recruited a community sample of 16 adults with self-identified chronic tinnitus from advertisements in local online newsletters and noticeboards. We also recruited 16 age- and sex-matched healthy controls from the community. We included right-handed people between the ages of 18–60 years and excluded people with significant neurological or psychological pathologies (*n* = 2, one participant from each group), resulting in a final matched sample of 15 people with chronic tinnitus and 15 matched controls (participant information is summarized in Table 1). The chronic tinnitus group were screened prior to recruitment to ensure they met the following criteria for chronic tinnitus: (1) experienced tinnitus for more than 3 months and (2) reported the tinnitus was always present^52^. The study was approved by the Human Research Ethics Committee of The University of Melbourne and was carried out in accordance with the Declaration of Helsinki. All participants gave written informed consent prior to participating.

**Table 1:** Participant Characteristics.

	Chronic Tinnitus (*n* = 15)	Healthy Controls (*n* = 15)
Age in years (SD)	36.73 (11.81)	36.13 (10.94)
Gender	60% female	60% female
Hearing impairment	73% none	100% none
	13% slight	
	13% moderate	
HADS-A	5.47 (3.20)	5.13 (2.83)
HADS-D	1.80 (1.61)	1.93 (1.71)

HADS-A = Anxiety subscale of the Hospital Anxiety and Depression Scale, HADS-D = Depression subscale of the Hospital Anxiety and Depression Scale.

We used the psychometrically valid Hospital Anxiety and Depression Scale (HADS) to screen for symptoms of anxiety and depression as an indicator of emotional wellbeing^53^ (Table 1). We obtained pure-tone audiograms from participants and used the World Health Organisation Hearing Impairment Scale to judge hearing health^54^. We used the *n*-back task to activate the CCN due to its established properties as a replicable and reliable task for imaging studies to optimise power in this study with our chosen sample size (*n* = 30), determined by funding availability and successful task activation observed in previous studies using this task and similar sample sizes^55–57^. We validated our sample size using G*Power (v3.1.9.2), which that estimated a total sample size of 30 would detect an effect size recently reported in an fMRI *n*-back study (*d* = 1.12^57^; with power of 0.75 for a corrected t-test with α = 0.025.

All controls and the majority of tinnitus participants (73%) had normal hearing, with mean audiograms for both groups showing hearing in the normal range. We found no significant hearing differences between the two groups (*p* = 0.099), and the mean hearing thresholds for each of the four main frequencies (500, 1000, 2000, 4000 Hz) was <25 dB in both groups, suggesting normal hearing at the group-level. In addition, both groups reported minimal symptoms of anxiety or depression on the HADS, with no significant group differences for emotional wellbeing on this measure, and no significant differences in age or gender (all *p* > 0.500). While all participants reported their tinnitus was ‘always’ present, qualitative report on the Tinnitus Sample Case History Questionnaire indicated they were aware of the sensation 46.33% of their awake time (SD = 24.16%; Table 2)^58^. These ratings highlight the distinction between the *presence* of tinnitus (constant) and their *awareness* of the sensation (half of their awake time). Consistent with this, most tinnitus participants (67%) reported a mild impact of their chronic tinnitus on daily life, the grading most commonly endorsed by people with chronic tinnitus, suggesting this is a representative sample of this population^46^ (tinnitus characteristics are summarized in Table 2).

**Table 2:** Tinnitus Characteristics.

Characteristic	Mean (SD)	n(%)
Time with tinnitus (years)	16.27 (13.94)	
Mean THI score (0–60)	28.80 (13.96)	
Slight impact		2 (13%)
Mild impact		10 (67%)
Moderate impact		2 (13%)
Severe impact		1 (7%)
Awareness (0–100)	46.33 (24.16)	
Loudness (0–100)	45.17 (21.64)	
Tinnitus laterality		
Left ear		1 (7%)
Both ears, worse in left		0 (0%)
Both ears/Inside the head		9 (60%)
Both ears, worse in right		3 (20%)
Right ear		2 (13%)
Onset		
Sudden		4 (27%)
Gradual		10 (67%)
Unknown		1 (7%)
Believed cause		
Known^a^		12 (80%)
Unknown		3 (20%)

^a^Causes believed to be prolonged noise exposure (33%), stress (20%), medical (20%), loud sound blast (20%), and hearing changes (7%).

### Cognitive Task

We used a visual version of the *n*-back task^59^, which was programmed using ‘Presentation’ software^60^. Participants completed two block-design paradigms where they had to respond to letters on screen as quickly and accurately as possible. Within each paradigm, we pseudorandomly alternated between three conditions: baseline, 0-back (low cognitive load), and 2-back (high cognitive load). In the 0-back condition, participants were instructed to respond every time a letter “X” appeared (25% of trials), where “X” was one of 15 possible capital letters. In the 2-back condition, participants were instructed to respond if the letter was the same as the letter seen two previously (25% of trials). The two paradigms used different baseline conditions, with one run using a passive baseline, where participants stared at a fixation cross (rest-baseline), and the other run using an active baseline, where participants responded to every letter (motor-baseline). Baseline conditions lasted 15 seconds and the cognitive conditions (low and high load) lasted 24 seconds each, with each run featuring 5 of each block type (baseline, low-load, high-load).

Stimuli were 15 capital letters in white font on a black background. The letters (“A”, “B”, “C”, “D”, “E”, “F”, “H”, “I”, “L”, “M”, “N”, “O”, “T”, “Y, “Z”) were shown in pseudorandom order and participants had to identify and efficiently respond to the target letter(s). Each letter appeared for 500 ms, followed by a black screen for 1000 ms (inter-stimulus interval = 1.5 seconds). Before each paradigm, participants were verbally reminded of the task instructions, and within each paradigm, each block was preceded by a task prompt (‘Rest’ (rest-baseline), ‘Detect-All’ (motor-baseline), ‘Detect-X’ (0-back), or ‘2-back’ (2-back)), which appeared on the screen for 3 seconds, and a small prompt remained in grey text below the task stimuli (“Rest”, “All’, “X’s”, or “2-back”, respectively). Training prior to the scan was done on a 13” laptop screen with responses made using a wireless mouse.

### Image Acquisition

Images were acquired on a 3 Tesla Siemens Skyra MRI scanner (Siemens, Erlanger, Germany). The blood oxygen level-dependent (BOLD) functional imaging parameters were as follows: 44 slices with 3 mm thickness, TR = 3000 ms, TE = 30 ms, flip angle = 85°, voxel size of 3 × 3 × 3 mm, and an acquisition matrix of 72 × 72. Additional pairs of images were collected with reversed phase-encode blips, resulting in pairs of images with distortions going in opposite directions. From these pairs, the susceptibility-induced off-resonance field was estimated using a method similar to that described by Andersson and colleagues^61^ as implemented in FSL^62^, enabling distortion correction of functional images. T1 weighted images were also acquired during the same session for co-registration to functional images. SPM12 (Wellcome Department of Imaging Neuroscience, London, http://www.fil.ion.ucl.ac.uk/spm/), FSL (FMRIB Software Library, University of Oxford, http://www.fmrib.ox.ac.uk/fsl), and MRtrix (http://www.mrtrix.org/) were used for preprocessing of functional images. The functional images were slice-time corrected (FSL’s *slicetimer* function), realigned (FSL’s *mcflirt* function), distortion corrected (FSL’s *topup* function), co-registered to the anatomical T1-weighted images (FSL’s *epi_reg* function), nonlinearly warped to Montreal Neurological Institute (MNI) space using the deformation field obtained by segmenting the T1 images (SPM12’s *segment* module), and spatially smoothed with a Gaussian kernel (FWHM = 8 mm).

### Procedure

After providing informed consent, we assessed participant hearing and collected demographic information. The chronic tinnitus group also provided a history of their tinnitus experience using the Tinnitus Case Sample History Questionnaire^58^, and we assessed the impact of their tinnitus on daily life using the gold standard Tinnitus Handicap Inventory (THI; tinnitus characteristics are summarised in Table 2)^63^. Participants were then trained on the cognitive control task until they were proficient and could successfully identify 66% of targets in the 2–back condition prior to the scan, in order to eliminate risk of poor performance influencing network activation. They then completed the task twice in the MRI scanner, providing two independent data sets for analysis. During performance of the cognitive task in the scanner, we recorded participant responses using a button-box in the right-hand. After the scanning session participant’s completed 10-point rating scales to assess (a) how intrusive the noise of the scanner was based on five descriptors of sound intrusiveness (annoying, painful, distressing, irritating, aggravating), (b) how well participants thought they performed on the cognitive control task, and (c) if tasks were harder in the scanner than during practice.

### Statistical Analysis

#### Behavioural Analysis

We conducted independent-samples t-tests to compare the two groups on demographic and emotional wellbeing information and ratings of task and scanner noise intrusiveness. We used analysis of variance (ANOVA) models to assess behavioral performance on the cognitive control task (mean reaction time) and subjective ratings of performance on each task. Where assumptions of normality were violated, we used non-parametric versions of the tests; however, as these tests showed the same results, we have reported the parametric statistical tests.

#### Imaging Analysis

We performed analyses addressing our first question about activation of the cognitive control network in SPM12. For first level analyses of task-related activation, the design matrix contained regressors of interest modelling “0-back” and “2-back” blocks, and a regressor modelling the cue periods, which was included to account for any noise associated with the cues. Regressors were constructed by convolving boxcar functions with the canonical hemodynamic response function (HRF). The design matrix also included the six motion parameters estimated during image realignment. Data were high-pass filtered with a cut-off of 182 s and pre-whitened to correct for autocorrelation (AR1) in the data. We constructed a contrast of parameter estimates coding [*2-back* – *0-back*].

#### Defining Regions of Interest

We used the motor-baseline version of the task to define regions of interest (ROI) for further analysis in the second (i.e. independent) administration of the task using rest-baseline to achieve a noncircular analysis^21^. To do this, we generated group level activation images by performing one sample t-tests on the con images produced from this run of the cognitive task (SPM-T images; see Fig. 1a). We formed ROIs by thresholding the control SPM-T image at a feature threshold of 0.00001, yielding two ROIs in the right middle frontal gyrus (rMFG). Average activations within these ROIs were then calculated for each participant from the [*2-back* – *0-back*] contrast on the rest-baseline version of the cognitive task (i.e., using independent data from the data used to form ROIs). Activations in each group were then compared using a between groups t-test (p < 0.025).

### Exploratory Analyses

For these analyses we hypothesised that (1) there may be dysfunctional connectivity from the salience network (SN) to the affected node of the cognitive control network (CCN; rMFG) and (2) that the affected CCN node would show dysfunctional connectivity with nodes of the autobiographical memory network (AMN), also known as the ‘task-negative’ network. To address this question we performed post-hoc psychophysiological interaction (PPI) analyses using a multiregional generalised PPI approach to investigate the task-dependent and task-independent (i.e. baseline) connectivity between nodes of the neurocognitive networks (AMN, CCN, SN) thought to be involved in chronic tinnitus and attention-switching^7,64–66^. This analysis assesses functional interactions between pairs of pre-selected ROIs or seeds^65^.

#### Defining Regions of Interest

To define these ROIs, we used de/activation maxima that fell within these regions in the SPM-T image of the [*2-back* – *0-back*] contrast from the motor-baseline task run in controls. For the first analysis this yielded 2 seed ROIs from the salience network: the anterior insula (*xyz* mm = [32, 24, 4]) and right anterior cingulate [6, 14, 48], and two target ROIs for the second analysis from the autobiographical memory network: left posterior cingulate cortex [−6, −48, 30], and left medial prefrontal cortex [−8, 60, 8]. The affected node of the CCN was consistent across all analyses (rMFG [46, 6, 28]). For each participant and region, brain activity (the first eigenvariate) from the motor-baseline cognitive task run was extracted from within a 5 mm radius sphere centred on the seed coordinate.

#### Psychophysiological Interactions

Using the gPPI approach^66^, a PPI vector for each region in each participant was generated for the “2-back”, “0-back”, and “cue” regressors (the task related regressors from participants’ first level activation analyses). These PPI vectors were calculated as the elementwise product of the seed region’s activity with the corresponding regressors. The HRF was deconvolved from the region’s activity prior to multiplication, and the result then convolved with the HRF to yield the final PPI vector (using *spm_peb_ppi.m*). The three PPI vectors were then entered into a general linear model (GLM), along with the seed region’s activity (i.e. baseline connectivity), and the HRF convolved “2-back”, “0-back”, and “cue” regressors. The activity of the region being influenced (i.e., the target) was the dependent variable. We compared connectivity between focused cognitive activity and rest by using a contrast with positive weights on the “2-back” and “0-back” PPI terms. This quantifies the change in the slope of the relationship between activity in the seed and target regions that occurs between the baseline (tap) and task-active (0- and 2-back) conditions. We used t-tests to compare the task-dependent and task-independent connectivity terms between the chronic tinnitus and matched control groups. While the task-dependent (PPI) terms failed to show significant differences between the groups, significant differences in baseline connectivity were identified.

#### Behavioural Correlates

We conducted Pearson correlations (*r*) to assess associations between significant connectivity differences, activation of the rMFG, scanner-intrusion and task difficulty. Where assumptions of parametric tests were not upheld, more conservative non-parametric Spearman correlations were conducted, however as no differences between parametric and non-parametric test outcomes were observed the results of parametric tests are reported. We computed 95% confidence intervals using bias corrected boot-strapped confidence intervals based on 1000 samples (BCa CI).
